# Results and DVH analysis of late rectal bleeding in patients treated with 3D-CRT or IMRT for localized prostate cancer

**DOI:** 10.1093/jrr/rru080

**Published:** 2014-09-11

**Authors:** Masanori Someya, Masakazu Hori, Kunihiko Tateoka, Kensei Nakata, Masaru Takagi, Masato Saito, Naoki Hirokawa, Masato Hareyama, Koh-ichi Sakata

**Affiliations:** 1 Department of Radiology, Sapporo Medical University School of Medicine, Chuo-ku, Sapporo, Hokkaido, 060–8543, Japan; 2 Department of Radiology, Hyogo Ion Beam Medical Center, 1-2-1 Koto, Shingu-cho, Tatsuno-shi, Hyogo, 679–5165, Japan; 3 Southern Tohoku Proton Therapy Center, 172–7, Yatsuyamada, Koriyama, Fukushima, 963–8563, Japan

**Keywords:** prostate cancer, 3D-CRT, IMRT, late rectal toxicity, DVH analysis

## Abstract

In patients undergoing radiotherapy for localized prostate cancer, dose–volume histograms and clinical variables were examined to search for correlations between radiation treatment planning parameters and late rectal bleeding. We analyzed 129 patients with localized prostate cancer who were managed from 2002 to 2010 at our institution. They were treated with 3D conformal radiation therapy (3D-CRT, 70 Gy/35 fractions, 55 patients) or intensity-modulated radiation therapy (IMRT, 76 Gy/38 fractions, 74 patients). All radiation treatment plans were retrospectively reconstructed, dose–volume histograms of the rectum were generated, and the doses delivered to the rectum were calculated. Time to rectal bleeding ranged from 9–53 months, with a median of 18.7 months. Of the 129 patients, 33 patients had Grade 1 bleeding and were treated with steroid suppositories, while 25 patients with Grade 2 bleeding received argon plasma laser coagulation therapy (APC). Three patients with Grade 3 bleeding required both APC and blood transfusion. The 5-year incidence rate of Grade 2 or 3 rectal bleeding was 21.8% for the 3D-CRT group and 21.6% for the IMRT group. Univariate analysis showed significant differences in the average values from V65 to V10 between Grades 0–1 and Grades 2–3. Multivariate analysis demonstrated that patients with V65 ≥ 17% had a significantly increased risk (*P* = 0.032) of Grade 2 or 3 rectal bleeding. Of the 28 patients of Grade 2 or 3 rectal bleeding, 17 patients (60.7%) were cured by a single session of APC, while the other 11 patients required two sessions. Thus, none of the patients had any further rectal bleeding after the second APC session.

## INTRODUCTION

When radiotherapy is performed for the treatment of localized prostate cancer, high doses need to be delivered to improve outcomes [1, 2]. However, an increase in the radiation dose used to treat prostate cancer is associated with a relatively high risk of rectal complications, and this problem has limited dose escalation. Development of 3D-CRT has allowed the high-dose volume to be shaped to the target, resulting in the irradiation of smaller doses of normal tissue and fewer treatment-related complications. The subsequent implementation of dose escalation with IMRT has resulted in enhanced local tumor control by increasing the dose without significant treatment-related morbidity [3–6]. However, a moderate increase in late rectal toxicity, mainly rectal bleeding, has been reported in association with such dose intensification, so the identification of parameters that can predict rectal bleeding is important [7–9]. In this study, dose–volume histograms (DVHs) and clinical variables were examined by univariate and multivariate analyses to search for correlations between radiation treatment planning parameters and late rectal bleeding.

## MATERIALS AND METHODS

Between February 2002 and September 2010, 129 patients with localized prostate cancer were treated at our institution. Of these, 55 patients were treated with 3D-CRT between February 2002 and May 2007, while 74 patients received IMRT between August 2007 and September 2010. All patients were confirmed to have adenocarcinoma of the prostate by biopsy. All patients received definitive radiotherapy with curative intent. Their risk groups were classified according to the guidelines of the National Comprehensive Cancer Network [10]. Patient characteristics are listed in Table 1. This study was approved by our institutional review board and written informed consent was obtained from all patients.

**Table 1.** Patient characteristics

	3D-CRT	IMRT	*P*-value
	(*n* = 55)	(*n* = 74)	
Age (mean)	55–79 (69.3)	49–84 (70.0)	0.589
NCCN risk group			
Low	4	9	
Intermediate	20	31	
High	24	27	
Very high	7	7	0.904
T1c	24	55	0.023
T2a	10	6	
T2b	6	2	
T2c	1	0	
T3a	7	4	
T3b	7	7	
Gleason score			
5–6	9	16	
7	35	29	
8–10	11	29	0.006
PSA (ng/ml)			
<10	17	35	
10–20	16	26	
>20	22	13	0.180
Hypertension	24	27	0.213
Diabetes mellitus	7	7	0.553
Usage of anticoagulants	6	17	0.154

### Treatment

For daily treatment, patients were requested to empty the rectum and urinate at 1 h before radiotherapy and then drink 500 ml of water in order to achieve a consistent state of the bladder and rectum. All radiotherapy involved the delivery of 10-MV X-rays with Linear accelerators.

3D-CRT was performed using a Varian Clinac 2100 (Varian Medical Systems, Inc., Palo Alto, CA), with FOCUS (Elekta AB, Stockholm, Sweden) being used for dose calculation in treatment planning. The dose was set as 70 Gy at the isocenter and was delivered in 35 fractions. The clinical target volume (CTV) was defined according to risk, as evaluated by the NCCN guideline. In the low- and intermediate-risk group, the CTV was defined as the prostate and the proximal portions of the seminal vesicles. In the high- and very high-risk groups, the CTV included the entire prostate and the seminal vesicles. The planning target volume (PTV) margin was set at 1 cm in all directions for 3D-CRT planning. Rotational conformal radiotherapy was employed.

IMRT was delivered with a Primus (Siemens AG, Munchen, Germany). From the start of IMRT, 3D-bone matching with an ExacTrac X-Ray 6D stereotactic image-guided radiation therapy system (BrainLAB AG, Feldkirchen, Germany) was introduced. In treatment planning, XiO ver. 4.62 (Elekta AB, Stockholm, Sweden) was used for dose calculation. The dose covering 95% of the target volume (D95) was set as 76 Gy and was delivered in 38 fractions. The CTV was defined according to risk, as evaluated by the NCCN guidelines. In the low-risk group, the CTV was defined as the prostate and the proximal portions of the seminal vesicles. In the intermediate-, high- and very high-risk groups, the CTV included the entire prostate and the seminal vesicles. The PTV margin was 8 mm in all directions except posteriorly, where it was 5 mm. Seven to nine planar beams were used.

### Hormonal therapy

Androgen deprivation therapy (ADT) was administered to 12 of the 51 intermediate-risk patients (23.5%) and 52 of the 65 high- and very high-risk patients (80.0%). ADT was commenced from 3–6 months prior to radiotherapy in 61 patients with a larger prostate volume (40 ml or more) or high risk, concurrent ADT was administered to 53 patients, and adjuvant ADT was administered for 2 or 3 years in 38 of the intermediate-, high- and very high-risk patients. Treatment details are listed in Table 2.

**Table 2.** Treatment characteristics

	3D-CRT	IMRT
	(*n* = 55)	(*n* = 74)
PTV mean dose (Gy)	65.4–72.0 (69.3)	71.6–80.3 (78.4)
RT to DSV	13	42
Prostate volume (ml)	16.4–65.4 (32.2)	16.5–75.4 (34.5)
Rectum volume (ml)	25.0–116.0 (52.9)	25.7–97.2 (51.6)
ADT Neoadjuvant	29	32
Concurrent	21	32
Adjuvant	17	21

Median values are showed in parentheses. DSV = distal portion of seminal vesicle, ADT = androgen deprivation therapy.

### Follow-up

After the completion of radiotherapy, follow-up evaluation was performed at 3–6-month intervals for 5 years and every 6 months thereafter. The median follow-up period for this study was 85 months (19–135 months) in the 3D-CRT group and 38 months (11–66 months) in the IMRT group. The cut-off date for analysis was August 2013.

### Evaluation of late rectal bleeding

Rectal bleeding was confirmed by digital rectal examination and endoscopy, and was graded with our institutional criteria (modified from the National Cancer Institute Common Terminology Criteria for Adverse Events ver. 4.0 [NCI-CTC AE 4.0]). All time intervals were measured from the completion of radiotherapy to the onset of rectal bleeding. Our institutional criteria for classification of rectal bleeding were as follows: Grade 0 was no bleeding, Grade 1 was mild bleeding that did not require intervention, Grade 2 was bleeding 2–3 times per week that required endoscopic cauterization, and Grade 3 was bleeding that required transfusion and/or surgical intervention.

For those who were lost to follow-up, grade of rectal bleeding was retrospectively scored by review of the medical record, and for those patients with regular follow-up, the score was reconfirmed by an interview on the last visit.

### DVH review

All treatment plans were retrospectively reconstructed using XiO and were reviewed by one of our authors, with minor adjustment of normal tissue contours being done as necessary to maintain consistency. The rectum was contoured as a solid organ from the level of the bilateral ischial tuberosities to the rectosigmoid junction. All DVHs of the rectum were regenerated. Then the maximum rectal dose (Rmax) and the relative volume doses delivered to the rectum (V75, 70, 65, 60, 55, 50, 45, 40, 30, 20 and 10) were calculated.

### Statistical methods

The Student *t*-test and the chi-square test were used to compare differences between two groups. Kaplan–Meier analysis was employed to determine the cumulative incidence of rectal bleeding. The Cox proportional hazards model was used to identify variables that had an influence on rectal bleeding. Stat View software ver. 4.58 (Abacus Concepts, Berkeley, CA) was employed for statistical calculations.

## RESULTS

### Rectal bleeding rate

**Fig. 1.**
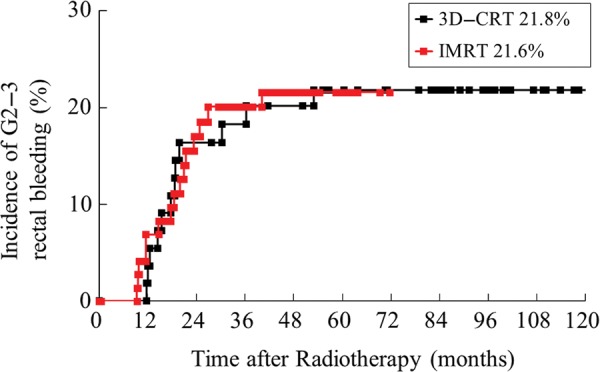
Incidence of Grade 2–3 rectal bleeding after radiotherapy. The 5-year incidence rate of Grade 2 or 3 rectal bleeding was 21.8% in the 3D-CRT group and 21.6% in the IMRT group.

The time to the onset of rectal bleeding ranged from 9–53 months, with a median of 18.7 months. In 33 patients, bleeding was classified as Grade 1 and they were treated with steroid suppositories. Another 25 patients had Grade 2 bleeding and received APC, while three patients with Grade 3 bleeding required both APC and blood transfusion. The 5-year incidence rate of Grade 2–3 rectal bleeding was 21.8% for the 3D-CRT group and 21.6% for the IMRT group (Fig. 1). In 17 of the 28 patients (60.7%) of Grade 2 or 3 rectal bleeding, rectal bleeding was cured by a single session of APC. The other 11 patients required two sessions of APC, and the interval between the first and second APC sessions was 1, 2, 3, 8, 11, 11, 11, 12, 20, 33 and 59 months, respectively. None of the patients had any further rectal bleeding after the second APC session.

**Fig. 2.**
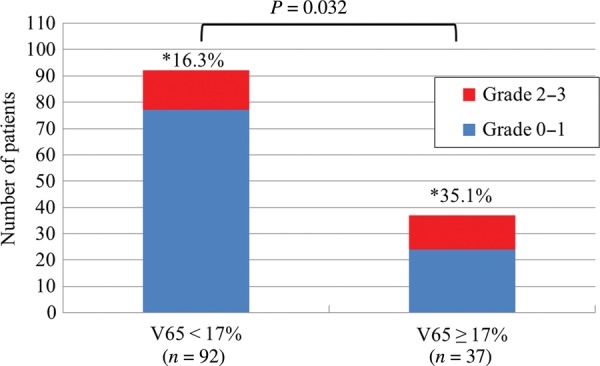
Relationship between V65 and rectal bleeding. When patients were divided into two groups (V65 < 17% or V65 ≥ 17%), Grade 2 or 3 rectal bleeding was more frequent in the group with V65 ≥ 17%. Asterisk = the percentage of Grade 2–3 rectal bleeding.

The relationship between the rectal radiation dose and rectal bleeding is displayed in Table 3. Univariate analysis showed that the average values of V65 and V10 were significantly different between Grades 0–1 and Grades 2–3. When the patients were divided into two groups with V65 < 17% or ≥ 17%, the rate of Grade 2 or 3 rectal bleeding was significantly higher in the V65 ≥ 17% group (16.3% for V65 < 17% vs 35.1% for V65 ≥ 17%) (*P* = 0.032, Fig. 2).

**Table 3.** Comparison of rectal dose and grade of bleeding

Variables	Grade 0–1	Grade 2–3	*P*-value
	(*n* = 101)	(*n* = 28)	
Rmax (Gy)	76.9 ± 6.7	77.6 ± 6.8	0.451
V75 (%)	5.1 ± 4.9	5.6 ± 5.3	0.695
V70	7.3 ± 6.7	9.7 ± 8.1	0.173
V65	15.0 ± 4.8	19.5 ± 8.2	0.007
V60	20.0 ± 6.3	24.9 ± 10.0	0.019
V55	24.3 ± 9.6	29.5 ± 12.0	0.042
V50	28.1 ± 10.6	33.8 ± 13.7	0.048
V45	32.0 ± 10.9	38.1 ± 14.6	0.048
V40	36.2 ± 11.2	42.2 ± 14.8	0.050
V30	46.5 ± 12.2	53.0 ± 14.1	0.033
V20	62.7 ± 14.0	68.2 ± 12.5	0.050
V10	80.2 ± 14.8	85.4 ± 12.0	0.050

Rmax = maximum dose to rectum. The *t*-test was used to compare between two groups.

The average values of V65 in patients with Grade 2 bleeding with a single session of APC (17 patients), Grade 2 bleeding with multiple sessions of APC (11 patients) and Grade 3 (three patients) was 18.8 ± 6.4, 20.1 ± 9.9 and 19.5 ± 8.5, respectively. There were no significant differences among the three groups.

### Prognostic factors according to multivariate analysis

The age, prostate volume, treatment method (3D-CRT or IMRT), presence of hypertension, presence of diabetes mellitus, use of anticoagulants, and rectal dose were assessed by multivariate analysis as potential risk factors for Grade 2 or 3 rectal bleeding. As a result, the rectal dose, especially V65 ≥ 17%, was found to be a significant risk factor for rectal bleeding (*P* = 0.038), while the other variables were not (Table 4).

**Table 4.** Multivariate analysis of predictive factors for rectal bleeding

Variable	Hazard ratio	95% C.I.	*P*-value
Age	1.036	0.998–1.074	0.062
3D-CRT vs IMRT	1.280	0.762–2.150	0.085
V65 (<17% vs ≥17%)	1.719	1.032–2.865	0.038
Prostate volume	0.991	0.974–1.010	0.354
ADT	0.879	0.561–1.380	0.576
Hypertension	1.066	0.702–1.621	0.764
Diabetes	1.081	0.567–2.060	0.813
Usage of Anticoagulant	1.430	0.816–2.507	0.211

ADT = androgen deprivation therapy, C.I. = confidence interval.

## DISCUSSION

There are two classifications of late rectal bleeding after radiation therapy, the Radiation Therapy Oncology Group (RTOG) late toxicity criteria [11] and the National Cancer Institute Common Toxicity Criteria Adverse Event (NCI-CTC AE), which differ with regard to their details. Previous reports about late rectal bleeding after radiation therapy have used one or other classification, leading to difficulty in interpretation of the results. We used NCI-CTC AE (ver. 4.0) with some modification in this study and divided the subjects into two groups, which were Grades 0–1 and Grades 2–3. The use of APC for rectal bleeding was classified as being applied for Grade 2 bleeding in our analysis, and transfusion combined with APC was classified as being applied for Grade 3 bleeding. In the RTOG classification, the use of APC is classified as Grade 3, and transfusion combined with APC is classified as Grade 4.

We found that Grade 2–3 late rectal bleeding occurred in 21.8% of patients treated with 3D-CRT and 21.6% of those receiving IMRT. When the patients were divided into two groups with V65 < 17% or ≥ 17%, Grade 2–3 rectal bleeding was seen in 16.3% of the V65 < 17% group versus 35.1% of the V65 ≥ 17% group (Fig. 2). The results of our study indicated that a significant correlation exists between DVH and late rectal bleeding. In other words, the risk of late rectal toxicity is correlated with the rectal volume exposed to high radiation doses for the treatment of localized prostate cancer. When we compared the two groups (Grades 0–1 with Grades 2–3), the average values from V65 to V10 were significantly different according to univariate analysis, while multivariate analysis confirmed that V65 was a significant prognostic factor for Grade 2–3 late rectal bleeding.

Kuban *et al*. compared the impact of 70 Gy versus 78 Gy on gastrointestinal (GI) toxicity in 301 patients receiving 3D-CRT. After a median follow-up period of 8.7 years, GI toxicity worse than RTOG Grade 2 was significantly more frequent in high-dose patients (28% vs 15%, *P* = 0.013). DVH analysis showed that the incidence of complications could be decreased significantly by reducing the rectal treatment volume. When <25% of the rectum was treated with >70 Gy of radiation, the incidence of Grade 2 or worse complications at 6 years post-treatment was markedly reduced to 16% compared with 46% when this dose–volume cut-off point was exceeded [1]. Pederson *et al*. reported that the incidence of NCI–CTC AE rectal toxicity ≥ Grade 2 was 5% in 296 consecutive patients treated with IMRT after a median follow-up period of 41 months. They found that 100% of men with a rectal V70 ≤ 10%, V65 ≤ 20%, and V40 ≤ 40% were free from rectal toxicity ≥ Grade 2, as were 92% of men with a rectal V70 ≤ 20%, V65 ≤ 40%, and V40 ≤ 80%, and 85% of men exceeding these criteria were also free from the toxicity [12]. Tomita *et al*. reported on IMRT with helical tomotherapy for prostate cancer. They found that the maximum rectal doses, V70, and V60 of the RTOG toxicity ≥ Grade 2 group were significantly higher than those of the ≤ Grade 1 toxicity group according to univariate analysis, while V40 and V20 had no significant relationship with late rectal toxicity [13]. Thus, the trend for the risk of late rectal toxicity to be correlated with the percentage of the rectum receiving high doses such as V70 or V60 at other institutions was in agreement with our results.

We had more patients with Grade 2 bleeding compared with recent reports treated with IMRT. In most patients treated with IMRT in this series, the prescribed dose of 76 Gy in 38 fractions was delivered to D95 of the PTV. In the 3D-CRT groups, the prescription dose was 70 Gy in 35 fractions at the isocenter. Mizowaki *et al*. reported in their multi-institutional surveillance that the D95 prescription policy resulted in significant dose escalations of up to 5% in maximum case compared with the mean dose prescription [14]. This potential dose escalation may have caused a higher rate of late rectal bleeding in our results. In addition to that, there is the possibility that anterior wall of the rectum might incidentally expand anteriorly as an intrafractional motion during radiotherapy when 3D bone matching (ExacTrac system) without using cone beam CT scan is employed. Another possible rationale for higher rates of Grade 2 toxicity is the treatment policy for rectal bleeding in our institution to undergo APC early. The early use of APC could create a good success rate for APC (Table 5) but higher rates of Grade 2 bleeding, since a patient treated with APC is automatically classified as having Grade 2 bleeding. In our series, 68 patients had Grade 0 rectal bleeding, 33 patients had Grade 1 bleeding, 25 patients had Grade 2 bleeding, and 3 patients had Grade 3 bleeding. Approximately half of our patients received APC for the treatment of rectal bleeding, while the frequency of APC in rectal bleeding after radiotherapy for prostate cancer was much lower in other studies. Takemoto *et al*. performed APC in 12 out of 64 patients with rectal bleeding, while Tomita *et al*. reported that 2 of 27 patients with no response to steroid suppositories received APC [13, 15].

**Table 5.** Comparison of results of APC for rectal bleeding

	Our series	Takemoto *et al*.
	(*n* = 129)	(*n* = 403)
Radiation proctitis		
Grade 0–1	101 (78.3%)	370 (91.8%)
Grade 2–3	28 (21.7%)	33 (8.2%)
Number of patients treated with APC	28 (21.7%)	12 (3.0%)
Treated with single session of APC	17 (60.7%)	7 (58%)
2–3 sessions of APC	11 (39.3%)	5 (42%)
Success rate (cessation of bleeding after APC)	100%	42%

APC = argon plasma laser coagulation.

Table 5 compares the results of APC for rectal bleeding between our series and Takemoto's series [15]. There were more patients with Grade 2–3 bleeding in our study. All patients were treated with IMRT in Takemoto's study, while ∼40% were treated with 3D-CRT in our study. The success rate (stopping of bleeding after APC) was 100% at our institution and was higher than that reported by Takemoto. We actively performed APC before rectal bleeding progressed. The early use of APC is related to the higher frequency of APC and may be related to the higher cure rate obtained with a single session of APC at our institution. There were no significant complications from APC. This outcome reflected the skill of the gastroenterologists at our institution, who employed optimal APC parameters based on swine rectum experiments and had considerable experience with APC [16].

We also examined whether there were any relationships between DVH and factors indicating the severity of rectal bleeding, such as the frequency of APC and transfusion. However, no relationships were identified, indicating that the severity of rectal bleeding may be influenced by biological factors, such as individual variation of radiosensitivity [17], rather than physical parameters.

In conclusion, we demonstrated the impact of clinical characteristics and DVH parameters on late rectal toxicity in 129 patients receiving 3D-CRT and IMRT for localized prostate cancer. Late Grade 2–3 rectal bleeding occurred in 21.8% of patients after 3D-CRT and in 21.6% after IMRT, but could be safely treated with APC. The rectal dose, especially V65 ≥ 17%, was a significant risk factor for rectal bleeding.

## FUNDING

The investigation was partly supported by a Grant-in Aid for Scientific Research from the Ministry of Education, Culture, Sports, Science and Technology, Japan (Grant number is 24591846). Funding to pay the Open Access publication charges for this article was provided by Grant-in Aid for Scientific Research from the Ministry of Education, Culture, Sports, Science and Technology, Japan (Grant number is 24591846).
